# Expression of Dicer and Its Related MiRNAs in the Progression of Prostate Cancer

**DOI:** 10.1371/journal.pone.0120159

**Published:** 2015-03-13

**Authors:** Xiaojie Bian, Yijun Shen, Guiming Zhang, Chenyuan Gu, Ying Cai, Chaofu Wang, Yiping Zhu, Yao Zhu, Hailiang Zhang, Bo Dai, Dingwei Ye

**Affiliations:** 1 Department of Urology, Fudan University Shanghai Cancer Center, Shanghai, China; 2 Department of Oncology, Shanghai Medical College, Fudan University, Shanghai, China; 3 Department of Pathology, Fudan University Shanghai Cancer Center, Shanghai, China; University of South Alabama Mitchell Cancer Institute, UNITED STATES

## Abstract

Dicer is aberrantly expressed in several types of malignancies. Cleaved by Dicer, the small noncoding microRNAs (miRNAs) are considered potential tools for the diagnosis and prognosis of cancer. This study investigated the expression of miRNAs thought to target Dicer. Expression of 1,205 human miRNAs and miRNA*s were examined in four patients with prostate cancer (PCa) by miRNA array in which the threshold was set as two-fold. Seventy-three miRNAs and miRNA*s were significantly down-regulated while 10 were up-regulated in PCa tissues compared with matched histologically normal glands. Of these, miR-29b-1, miR-200a, miR-370, and miR-31, which were the most down/up-regulated and closely potentially target to the Dicer 3′ UTR, were investigated further. Tissues of primary tumors and matched normal prostate glands from 185 patients with PCa were collected for further investigation. Dicer mRNA levels were negatively correlated with miR-29b-1 (ρs = −0.177, p = 0.017), miR-200a (ρs = -0.489, p < 0.0001) and miR-31 (ρs = −0.314, p < 0.0001) expression. Compared with adjacent normal glands, PCa tissues showed significantly lower miR-200a and miR-31 expression levels. Furthermore, in metastatic PCa, the expression levels of miR-200a, miR-370, and miR-31 were dramatically higher than in localized PCa. Additionally, elevated expression levels of miR-200a and miR-31 appeared to be associated with castration-resistant PCa. These findings suggest possibilities that miR-200a and miR-31 target Dicer and are involved in the carcinogenesis, migration, and behavior of castration-resistant PCa, indicating that they could be potential biomarkers for monitoring PCa progression.

## Introduction

Prostate cancer (PCa) is currently the second leading cause of cancer-related death worldwide, and PCa-specific mortality is rising in many Asian countries [1]. Advanced or metastatic PCa remains incurable and androgen ablation only offers affected patients a median progression-free time of 2 years [2].

Many genetic factors such as polymorphisms and epigenetic alterations are thought to contribute to carcinogenesis and the progression of prostate adenocarcinoma. Moreover, several studies have investigated the roles of microRNAs (miRNA) in the initiation and progression of human cancers, leading to the exploration of new mechanisms for tumor development [3]. In novel synthesis pathway, long miRNAs precursors are transcribed by RNA polymerase II and then processed into mature miRNAs by the sequential action of Dicer and Drosha endonucleases. Argo2, a member of the Argonaute family of proteins, binds the miRNA and forms an RNA-induced silencing complex through complementary pairing with the target site on the 3′-untranslated region (UTR) of target mRNA to trigger either translational repression or mRNA degradation [4, 5]. Loss of miRNA biosynthesis, as seen in Dicer knockouts, is regarded as lethal. Recently, a Dicer-independent miRNA biogenesis pathway has been revealed, demonstrating a novel role of Ago protein in gene silencing [6]. Furthermore, some mature miRNAs can inversely regulate Dicer expression by combining with the Dicer 3′UTR [7].

Several studies have demonstrated the prognostic values of aberrantly expressed Dicer in ovarian, breast, colorectal, and bladder cancers [8–11]. Dicer has previously been shown to be up-regulated in PCa, although a lower expression of Dicer contributed to a shorter recurrence time [12]. The increased Dicer levels in PCa correlated with clinical stage, lymph node status, and Gleason score. Its up-regulation may also explain the global increase of miRNAs expression observed in PCa [12, 13]. In a *Pten* null mouse model for PCa, the complete ablation of Dicer activity significantly halted tumor growth and progression, demonstrating that miRNAs have critical roles in maintaining cancer cell fitness [14].

To better understand the relationship between miRNAs and Dicer in PCa development and progression, comprehensive analysis of the expression of miRNAs is necessary. The present study therefore explored differentially expressed miRNAs that have the potential to target the Dicer 3′ UTR with the aim of examining the relationship between miRNAs and Dicer in the continuous development of PCa.

## Materials and Methods

### Patient cohort

This study was approved by the review board of Fudan University Shanghai Cancer Center and an approved and signed review board informed consent form was obtained for each participant. Tissues of primary tumors and matched normal prostate glands were collected from 185 patients with PCa at the Fudan University Shanghai Cancer Center between 2007 and 2011. Of these, 144 patients underwent radical prostatectomy whereas 41 were administrated transurethral resection of the prostate. Clinical characteristics including age, pathological stage, Gleason score, androgen-dependent state, and initial prostate-specific antigen (PSA) levels have been stated in our previous work [15].

### RNA extraction and miRNA microarray

Total RNA was extracted from frozen tissue stored at −80°C using the total RNA extraction kit (Ambion Inc., Austin, TX) according to the manufacturer’s instructions. The RNA concentration and integrity were measured using the NanoDrop Spectrophotometer (ND-1000, Nanodrop Technologies, Wilmington, DE) and electrophoresis, respectively. Equal amounts (300 ng) of RNA from each patient and control were pooled and presented as two groups (primary tumor and corresponding normal prostate glands). MiRNA signature profiles were generated from the above two groups. Agilent microRNA arrays (v16.0, 8 × 60K, Agilent Technologies, Santa Clara, CA), containing 1,205 capture probes, were used to quantify genome-wide miRNAs expression in four pairs of primary tumor and matched normal prostate glands. The microarray work was performed using an Agilent microRNA array which was scanned by an Agilent Scanner G2505B (Agilent Technologies, Santa Clara, CA) and the acquired array images were analyzed by Agilent Feature Extraction software (version 10.7.3.1; Agilent Technologies, Santa Clara, CA). Instead of using a global median method, normalization and subsequent data processing were performed using the GeneSpring GX v11.5.1 software package (Agilent Technologies, Santa Clara, CA), in which the threshold was set as two-fold. Computational miRNA target prediction algorithms TargetScan (http://www.targetscan.org) and DIANA-microT (http://diana.imis.athena-innovation.gr/) were used to investigate the series of miRNAs potentially targeted to Dicer.

### Reverse transcription-PCR and real-time quantitative PCR

Total RNA (2 μg) was reverse transcribed using the RevertAid First Strand cDNA Synthesis Kit (Thermo Scientific, Lafayette, CA) according to the manufacturer’s instructions without an RNase inhibitor in a final volume of 20 μl at 25°C for 10 min, then 37°C for 60 min, followed by 70°C for 5 min. For miRNA analysis, the MicroRNA Reverse Transcription Kit (Haoqinbio Inc., Shanghai, China) with specialized primers was used according to the manufacturer’ s protocol at 25°C for 5 min, then 42°C for 45 min, followed by 85°C for 5 min.

Real-time quantitative (q)PCR was carried out using the ABI PRISM 7900 system (Applied Biosystems, Foster City, CA). TaqMan probes and primers (Roche, Basel, Switzerland) were used according to the manufacturer’s instructions. Total RNA (2 μg) was converted into cDNA according to the manufacturer’s protocol, and PCR was performed in a total reaction volume of 10 μl, including 5 μl TaqMan Premix Ex Taq (2×), 0.5 μl probe and primer premix (20×), 1 μl cDNA template, and 3.5 μl of double-distilled water. Reaction conditions included an initial denaturation step at 95°C for 10 min followed by 40 cycles of 95°C for 10 s and 60°C for 60 s. All experiments were performed in triplicate. For normalization, β-actin and U6 were used for Dicer and miRNAs, respectively. The mean of each triplicate was used to calculate relative concentrations (Dicer ΔCt = Ct mean Dicer—Ct mean β-actin; miRNAsΔCt = Ct mean miRNAs—Ct mean U6). Expression fold changes were calculated using 2^−ΔΔCt^ methods.

### Statistical analysis

Statistical analysis was performed using IBM SPSS version 20.0 (SPSS Inc., Chicago, IL). Normalized expression levels of Dicer and miRNAs underwent a normal distribution test. Differences in continuous variables were assessed using the *t-*test. Spearman’s rank correlation coefficient ρ_s_ was used to evaluate the association between normalized levels of Dicer and miRNAs or the Gleason score. All tests were two-sided using a significance level 0.05.

## Results

### MiRNA array and real-time qPCR validation

**Fig 1:**
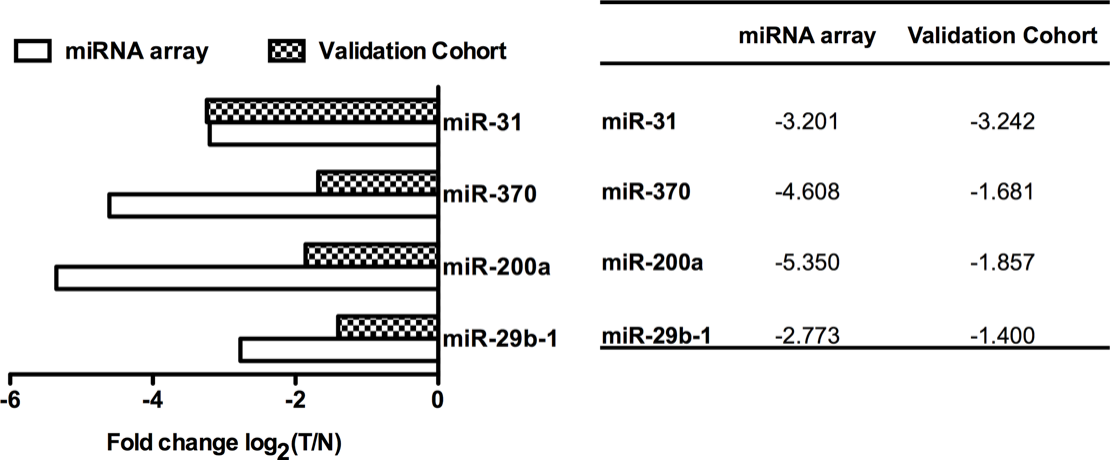
The comparison between miRNA array data and Real-Time qPCR results. For the comparison between miRNA array data and Real-Time qPCR results, miR-29b-1, miR-200a, miR-370 and miR-31determined to be differentially expressed in prostate adenocarcinoma compared to matched histologically normal glands in four patients by miRNA array were validated using Real-Time qPCR. The lengths of the columns in the chart represent the log2-transformed median fold changes (tumor/normal) in expression across the four patients for each of the four miRNAs validated.

We identified 73 miRNAs and miRNA*s that were significantly down-regulated and 10 that were up-regulated in PCa patients, which have the potential to be used to discriminate prostate adenocarcinoma from matched normal glands. MiR-29b-1, miR-200a, miR-370, and miR-31were selected for further validation of microarray results using real-time qPCR because they were not only the most down/up-regulated but also closely potentially target to the Dicer 3′ UTR. Data analysis showed that miR-29b-1, miR-200a, miR-370, and miR-31 were down-regulated in prostate cancer samples compared with matched normal tissues. The real-time qPCR results were consistent with those of microarray (Fig. 1).

### Dicer mRNA levels in PCa

**Fig 2:**
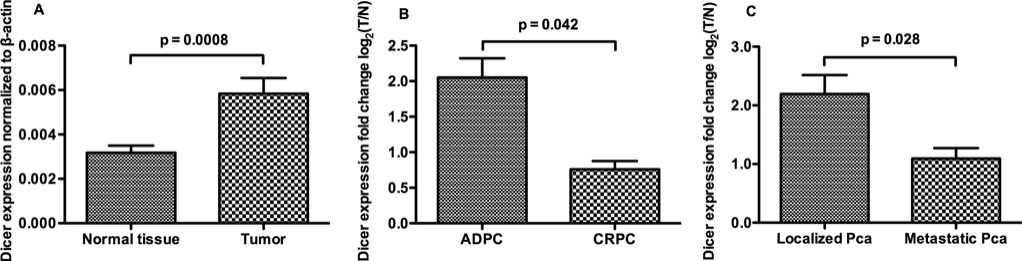
Expression of Dicer in PCa. **A,** Expression of Dicer in prostate adenocarcinoma and its matched normal glands, after normalization to β-actin; **B,** Expression of Dicer in androgen dependent and androgen independent PCa, fold changes of Dicer mRNA levels in prostatic adenocarcinoma versus matched normal glands were log2 transformed on Y axis; **C,** Expression of Dicer in localized and metastatic PCa, fold changes of Dicer mRNA levels in prostatic adenocarcinoma versus matched normal glands were log2 ranked on Y axis.

Dicer mRNA levels differed significantly between primary PCa tissues and matched normal tissues from 185 PCa patients (Fig. 2A). Moreover, compared with androgen-dependent PCa, the expression of Dicer was significantly lower in castration-resistant PCa (Fig. 2B), and in metastatic PCa when compared with the localized group (Fig. 2C). However, no significant correlations were observed between the Dicer expression and the total Gleason score (ρ_s_ = -0.082, p = 0.267) or main Gleason score (ρ_s_ = -0.054, p = 0.468).

### Differentiated expression of miRNAs in the validation cohort

**Fig 3:**
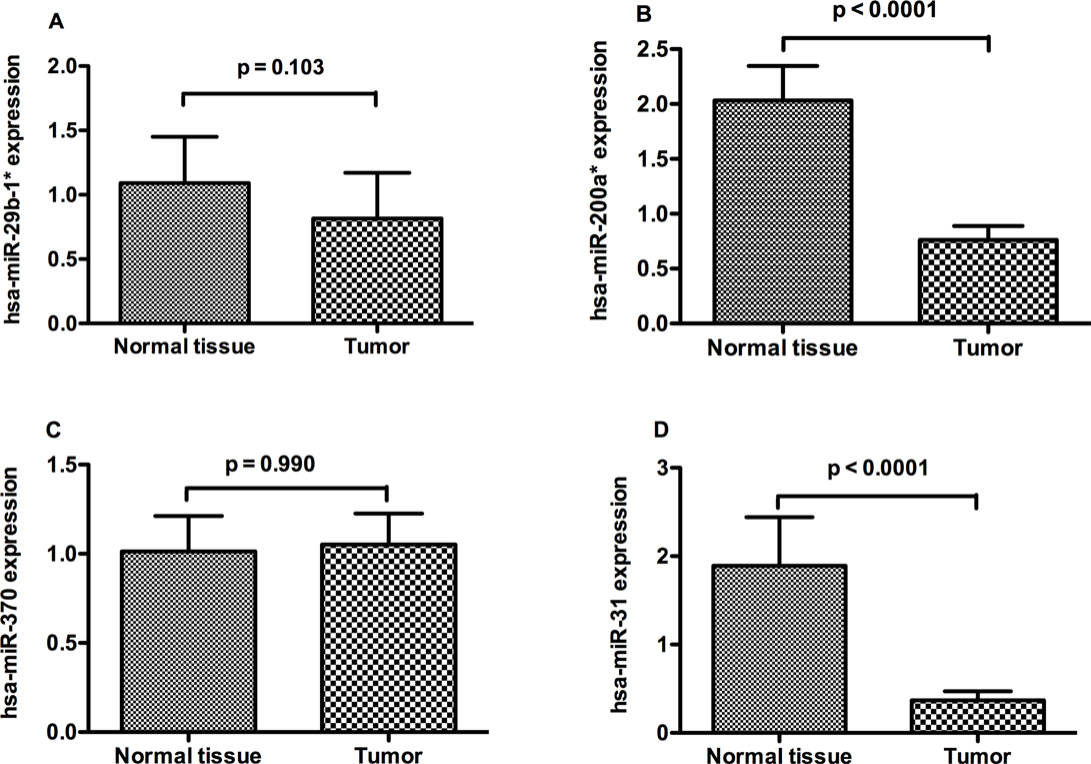
MiRNAs levels in validation cohort. Expression of miR-29b-1 (A), miR-200a (B), miR-370 (C) and miR-31 (D) in prostate adenocarcinoma and matched normal glands, after normalization to U6.

The expression levels of miR-200a and miR-31 was significantly down-regulated in PCa tissues compared with matched normal glands (Fig. 3B and 3D), while there was no significant difference in the expression levels of miR-29b-1 or miR-370 (Fig. 3A and 3C).

**Fig 4:**
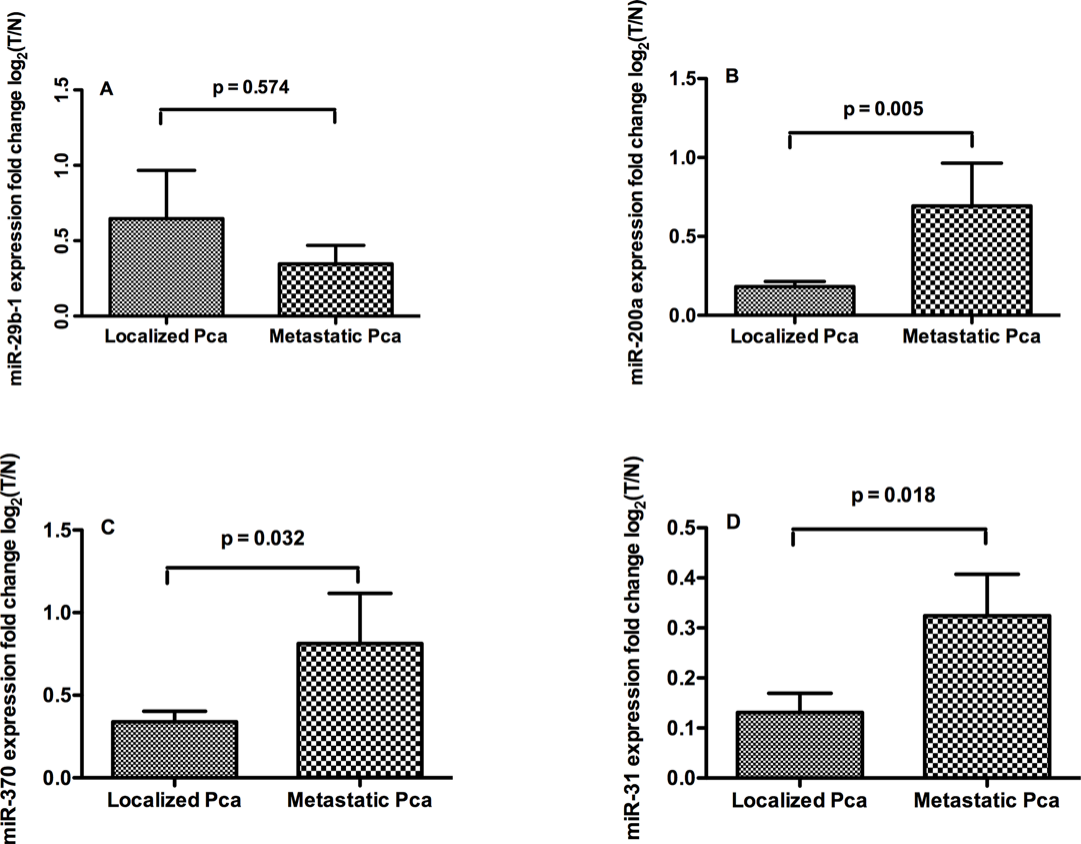
Comparison of miRNAs levels in localized and metastatic PCa. Expression of miR-29b-1 (A), miR-200a (B), miR-370 (C) and miR-31 (D) in localized and metastatic PCa, fold changes of miRNAs levels in prostatic adenocarcinoma versus matched normal glands were log2 transformed on Y axis.

Following the log2-transformation of miRNA fold changes in prostatic adenocarcinoma versus matched normal glands, the relative expression levels of miR-200a, miR-370, and miR-31 was higher in metastatic PCa compared with localized PCa (Fig. 4).

**Fig 5:**
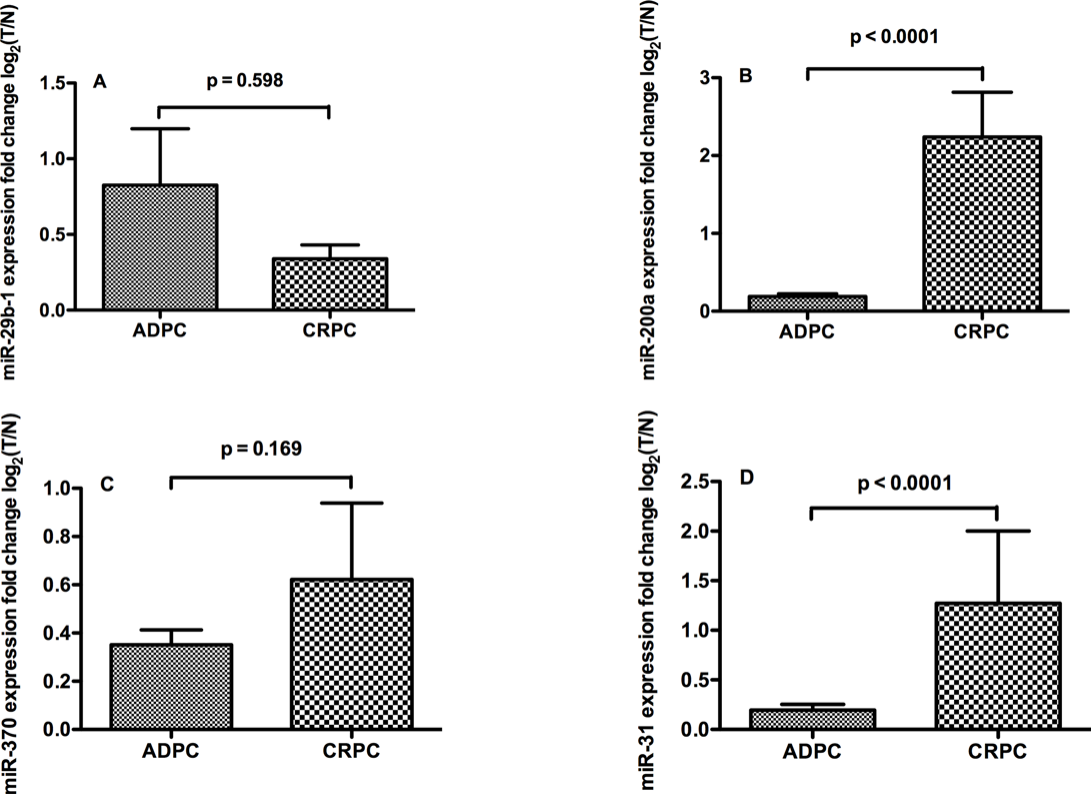
Discrimination of miRNAs levels in in androgen dependent and castration resistant PCa. Expression of miR-29b-1(A), miR-200a(B), miR-370(C) and miR-31(D) in androgen dependent and castration resistant PCa, fold changes of miRNAs levels in prostatic adenocarcinoma versus matched normal glands were log2 transformed on Y axis.

Cohorts were also divided into two subgroups according to their androgen-dependency, with 166 patients being androgen-dependent and 19 castration-resistant. Following log2 transformation of fold changes in prostatic adenocarcinoma versus matched normal glands, the relative expression levels of miR-200a and miR-31 was significantly lower in the androgen-dependent subgroup compared with the castration-resistant one (Fig. 5).

### Dicer mRNA in relation to miRNA expression

Correlation studies between Dicer and miR-29b-1, miR-200a, miR-370, and miR-31 expression in PCas showed that the expression of Dicer mRNA was moderately and negatively correlated with that of miR-200a and miR-31 (ρ_s_ = -0.489, p < 0.0001; ρ_s_ = -0.314, p < 0.0001, respectively). Besides, level of Dicer mRNA was found mildly and negatively correlated with miR-29b-1 (ρs = -0.177, p = 0.017) expression. However, there was no statistical association with the expression of Dicer to miR-31 expression levels (ρ_s_ = -0.057, p = 0.458) (Table 1).

**Table 1:** Correlations between Dicer mRNA and the miRNA expression levels in PCa.

miRNA	ρ_s_	p value
miR-29b-1	-0.177	0.017
miR-200a	-0.489	<0.0001
miR-370	-0.057	0.458
miR-31	-0.314	<0.0001

## Discussion

PCa is one of the leading causes of cancer-related death, and diverse mechanisms of PCa carcinogenesis and progression have been investigated. Several studies have demonstrated that miRNAs play important roles in the program by promoting degradation of the target gene. Although many miRNAs are known to be deregulated in cancers, it is unclear which have a pathogenic role in its development. Changes in miRNAs expression in cancer cell lines directly regulate their behaviors, such as proliferation and apoptosis [3], as well as influencing tumor suppression and metastasis. In the present study, we examined the expression levels of miR-29b-1, miR-200a, miR-370, and miR-31 in matched PCa tissues from 185 patients.

Previous studies have detected elevated levels of Dicer in primary PCa. We found that Dicer mRNA levels were significantly decreased in metastatic and castration-resistant PCa, which are associated with poor prognoses. Silencing Dicer could contribute to the activation of *p*-Akt and enhanced expression of cell cycle-associated molecules and proteins involved in tumor cell invasion. Conversely, the knock-down of Dicer was also shown to inhibit cell proliferation and promote apoptosis in leukemia cell lines [16]. These discrepancies could be explained by the observed heterogeneity and genetic diversity in different PCa tissues [17].

Our miRNA correlation study showed that the relative expression of miR-200a and miR-31 was moderately and negatively associated with Dicer mRNA levels in PCa. This result suggests a likelihood of a direct correlation between the miRNAs and Dicer because the miRNAs inhibit protein synthesis to preserve the stability of their mRNA target [4]. Comparing the expression levels of miR-200a and miR-31 between primary tumor and adjacent normal glands in the present study, we observed a significant expression decrease in primary tumors, suggesting that miR-200a and miR-31 may be involved in prostate carcinogenesis. Previous studies demonstrated that the miR-200 family is a crucial modulator of epithelial to mesenchymal transition, and that it is down-regulated and exhibits tumor suppressive properties in renal, prostate, breast, bladder, pancreatic, and gastric cancers [3, 18]. Additionally, the miR-200 family was shown to modulate the oxidative stress response, thus affecting tumorigenesis and chemosensitivity [19]. We found that miR-31 expression level was decreased in tumor tissues compared with normal glands, indicating that miR-31 may function as a tumor suppresser gene. However, the role of miR-31 remains controversial [20–22]. Chan et al. previously found that only one of three miR-31 isoforms, which differed only slightly in their 5′- and/or 3′-end sequences, directly repressed Dicer expression at both mRNA and translational levels in MCF-7 breast cancer cells and A549 lung cancer cells. This resulted in enhanced cellular sensitivity to cisplatin, a known attribute of Dicer knockdown [23].

During disease progression, miRNAs are thought to be prognostic factors for metastasis. We showed that miR-200a, miR-31, and miR-370 expression levels were increased in metastatic PCa rather than in localized cancers. *In vitro*, the transient transfection of miR-200a was previously shown to significantly reduce PCa cell line proliferation but have no effect on invasiveness [24]. In ovarian cancer stem cells, loss of miR-200a expression was thought to play a critical role in E-cadherin repression, thereby enhancing the migration and invasive abilities of CD133/1^+^ cells [25]. Moreover, miR-200a down-regulation in meningiomas promoted tumor growth by reducing E-cadherin expression and activating the Wnt/β-Catenin signaling pathway [26].

Similarly, a role for miR-370 in the proliferation of human PCa cells has previously been demonstrated. The ectopic expression of miR-370 promoted cell proliferation and increased the anchorage-independent growth and colony formation ability of DU145 and LNCaP PCa cells [27]. Higher expression of miR-370 in gastric cancer tissues was also associated with more advanced nodal metastasis and a higher clinical stage compared with controls, while patients with more advanced or invasive tumors demonstrated higher serum miR-370 expression levels. *In vitro* assays indicated that exogenous miR-370 expression enhanced the oncogenic potential of gastric carcinoma cells, targeted predicted sites in the transforming growth factor-b receptor II gene 3′UTR, and increased abdominal disseminated metastasis in nude mice [28]. In our study, miR-31 was elevated in metastatic PCa. Inhibition of miR-31 could promote *in vivo* metastasis through directly regulating a cohort of prometastatic genes in breast and ovarian cancers [29, 30]. Although miR-31 overexpression in a number of ovarian cancer cell lines with a dysfunctional TP53 pathway inhibited proliferation and induced apoptosis [30], its overexpression in the colon cancer cell line HT29, carrying a TP53 mutation, resulted in a strong anti-apoptotic effect. The anti-apoptotic effect of miR-31 over-expression was lower or absent in cells with a functional TP53 pathway [31]. Taken together, both the expression and functions of miR-31 appear to be cancer-specific and possibly cell context-dependent.

Castration resistance is a specific stage of advanced PCa associated with the failure of hormonal therapy and a poor prognosis. In our cohort, the expression levels of miR-200a and miR-31 were higher in primary tumors of castration-resistant PCa compared with androgen-dependent PCa, and higher in primary tumors compared with paired normal glands. This suggests a possibility that the up-regulated expression of miR-200a and miR-31 during carcinogenesis plays an important role in the development of PCa by promoting the cell into the castration stage. Several studies have already demonstrated that androgen receptor signaling plays a critical role in PCa pathogenesis. Recently, miRNAs were found to be mediators of androgen activity in the prostate and muscle, indicating that they may be important in the development of PCa [32]. Lin et al. found that miR-31 expression was reduced as a result of promoter hypermethylation in primary and metastatic PCa, and that it was inversely correlated with the aggressiveness of the disease. It was shown to directly target the androgen receptor at a site within the coding region commonly mutated in PCa [33]. Thus, the up-regulation of miR-31 may be one reason for the failure of androgen deprivation therapy. Additionally, up-regulated miR-31 could suppress cell cycle regulators and prevent cytotoxicity agents inducing apoptosis in PCa cells, making castration-resistant PCa therapy difficult [33, 34].

The role of elevated Dicer levels in the change from primary to metastatic and castration-resistant PCa tumors, as well as in relation to miRNAs, needs to be further explored in a larger cohort of patients. Our study did not continuously monitor these changes in each cohort, and the metastatic and castration-resistant samples were independent from the localized primary cancer tissues, so our findings are limited. Nevertheless, we believe that this pilot study was important to gain insights into how miRNAs might be regulated in metastatic and castration-resistant PCa patients. MiRNAs are emerging as a novel set of molecules that may ultimately lead to cancer formation when deregulated. They are also likely to cooperate with other classic oncogenes and/or down-regulate tumor suppressor genes to affect tumor behavior. These non-coding classes of RNA can serve as useful biomarkers and may greatly improve clinical management by enabling us to select more appropriate treatment options for patients [35]. Although major breakthroughs in translational oncology have been made in recent years, many issues remain to be addressed in patient management. Moreover, the mortality decline in the ‘PSA era’ is contentious because of PSA-based screening programs that also lead to overdiagnosis and overtreatment. Thus, novel and versatile molecules able to drive the decision-making process throughout the course of the disease are urgently needed.

In conclusion, our findings showed that Dicer might be a possible target for miR-200a and miR-31. Furthermore, these miRNAs might involve in the carcinogenesis, migration, and behavior of castration-resistant PCa, and could be used as potential biomarkers in monitoring the disease progression of PCa.
